# The integrative relationship between work–family conflict and turnover intention: a meta-analysis

**DOI:** 10.3389/fpubh.2025.1642843

**Published:** 2025-09-01

**Authors:** Guanghua Li, Yuhan Zhou, Xinyue Zhang, Igor Martek

**Affiliations:** ^1^College of Environment and Civil Engineering, Chengdu University of Technology, Chengdu, China; ^2^Sichuan Business School, Sichuan University, Chengdu, China; ^3^Chongqing City Vocational College, Chongqing, China; ^4^School of Architecture and Built Environment, Deakin University, Geelong, VIC, Australia

**Keywords:** meta-analysis, work–family conflict, work-to-family conflict, family-to-work conflict, turnover intention, moderating effects

## Abstract

The impact of work–family conflict on employee turnover intention has become a significant topic in organizational behavior research. However, existing findings show significant discrepancies, lacking systematic quantitative integration. The purpose of this study is to explore the relationships between work–family conflict, work-to-family conflict, family-to-work conflict, and employee turnover intention. It considers the moderating effects of national culture, occupation type, age, gender, and measurement tools. Methodology utilizes meta-analysis of large sample data, based on 122 empirical studies. It is found that: (1) There is a significant positive correlation between overall work–family conflict and turnover intention (r = 0.446), with both work-to-family conflict (r = 0.318) and family-to-work conflict (r = 0.261); (2) National culture (individualism/collectivism), occupation type, and measurement tool differences significantly moderate these three relationships; (3) Age only moderates the relationship between family-to-work conflict and turnover intention, while gender moderation effects are not supported. These findings enrich the knowledge system of turnover intention research and provide practical guidance on the implementation of effective measures to reduce turnover.

## 1 Introduction

Current economic environments are highly volatile, with employee turnover emerging as a major challenge for organizations (1). Significant attention has been paid by organizations to the phenomenon of voluntary turnover, as such behavior results in significant economic losses (2). High turnover rates have negative impacts on enterprise performance, manifesting as decreased productivity, reduced profits, increased recruitment costs, combined with the associated expenses of training new employees (2, 3). Employee turnover not only results in the loss of professional knowledge and skills, but also disrupts team collaboration, leading to increased workload and decreased morale (4). Empirical research has found that turnover intention is the most direct predictor of voluntary turnover behavior (5). Thus, an understanding of turnover intention can play a preventive role in the process of employee turnover management (6). Turnover intention refers to an individual’s assessment of the possibility of permanently leaving an organization at some future point, resulting in a weakening of employee organizational commitment and reinforcement of turnover intention (7).

The formation of employee turnover intention is influenced by multiple factors. Based on existing theories and literature, the influencing factors of employee turnover intention include not only macroeconomic environmental factors but also micro-level company factors, family factors, and individual factors. To a certain extent, the higher the level of economic development, the more employees are likely to consider job selection from the view of their developmental needs. Relevant research shows that the greater the job opportunities, or the better the employment situation, the greater the prevalence of employees forming a turnover intention (8). Moreover, work pressure can significantly predict employee turnover intention (9). Employee workload (10), interpersonal relationship depletion (11), work schedule conflicts, and work–family conflicts (12) also have significant positive impacts on employee turnover intention. Employees with higher family support have significantly lower turnover intentions even when facing high work demands (13). Research shows that individual variables such as age, gender, and education level indirectly affect employees’ turnover intentions (14). Of these, work–family conflict is a core influencing factor of turnover intention (15, 16). Analysis of the relationship between turnover intention and work–family conflict, as well as its influencing factors, can provide strategies for reducing employee turnover behavior in organizations.

Over the past few decades, the body of literature on work–family conflict has been growing steadily (17). It is now a significant theme in the field of organizational behavior and human resource management. Work–family conflict describes the social contradictions and conflicts that arise due to the pressures brought by persons having to perform multiple roles across the two domains of work and family (18). Indeed, employees increasingly experience excessive work-related and family-related conflicts (19).

Initially, researchers considered work–family conflict as a unidimensional construct (20). However, with the development and deepening of research, researchers have recognized that the conflict between work and family actually involves two interrelated but slightly different concepts (21). These are: work-to-family conflict and family-to-work conflict. According to the Conservation of Resources theory, when work responsibilities interfere with family life, a tendency for work-to-family conflict will emerge. Conversely, when family life interferes with work responsibilities, a tendency for family-to-work conflict eventuates (22). Each vector encompasses three forms of expression: time, pressure, and behavior (23). Time conflict manifests as contradictions in time allocation for role investment. Pressure conflict manifests as emotional spillover of stress from one domain to another. Behavior conflict stems from differences in role behavior expectations across different domains.

Most researchers in this area focus on the conflict between work and family domains, emphasizing how work–family conflict can affect individuals’ physical and mental health. Work–family conflict has become an increasingly common and prominent issue in the workplace (24). It can lead to negative outcomes such as decreased affective organizational commitment, increased absenteeism, and higher turnover intentions among employees (25).

Moreover, relevant research findings indicate that work–family conflict not only significantly affects work-related outcome variables but also exerts noticeable interference effects on family-related outcome variables. It can also affect outcome variables that are not domain-specific. Of these, work-related outcome variables include burnout (26), absenteeism (27), turnover intention (28), lower job performance, and job dissatisfaction. Frone et al. (29) proposed that work–family conflict tends to lead to poor job performance. Eby et al. (30) suggested that conflict is inversely proportional to individual job satisfaction while having a positive impact on turnover rate and absenteeism. By reducing the occurrence of work–family conflict, mitigating the negative effects of conflict, and strengthening individuals’ perceived organizational support, the goal of reducing employees’ counterproductive behaviors can be advanced (31).

Family-related outcome variables include family satisfaction (32), family labor participation, and marital satisfaction. The study conducted by Cerrato and Cifre (33) indicated that work–family conflict reduces individual time available for family labor participation through the “time allocation strain” mechanism. Here, women are more significantly affected due to traditional gender role expectations. Yoo et al. (34) found that family-to-work conflict indirectly reduces marital satisfaction through gender role beliefs. Non-specific outcome variables include emotional exhaustion, while work–family conflict directly and positively affects emotional exhaustion (35).

Some studies have also examined either mediating or moderating variables in the relationship between work–family conflict and turnover intention. For example, job burnout (36), work stress (37), emotional exhaustion (38), and organizational commitment (39) are considered mediating factors, while organizational support (40), national cultural values (41), and gender (42) are considered moderating factors.

With regards to the relationship between work–family conflict and turnover intention, various scholars have drawn diverse conclusions, with no unified conclusion being reached. Greenhaus et al. (25) found that work–family conflict significantly positively affects employees’ turnover intention, while also concluding that family–work conflict does not show the same intention. Wang et al. (43) agreed that work–family conflict has a significant positive impact on turnover intention. However, Wilkinson et al. (44) found that both work–family conflict and family–work conflict significantly positively affect turnover intention. Li et al. (39) found that work–family conflict has a significant impact on turnover intention, while the impact of family–work conflict did not reach a significant level. Added to this, Dan et al. (45) found that family–work conflict has a positive and significant direct effect on employees’ turnover intention, yet work–family conflict has no significant impact. Huang et al. (37) concluded that work–family conflict has no significant direct impact on turnover intention, but does indirectly affect it through work stress. They also confirmed that family–work conflict has a direct positive impact on turnover intention (37).

Meta-analysis is a quantitative research tool that can integrate and comprehensively analyze multiple studies on the same topic, leading to comprehensive conclusions. Although existing studies have constructed multi-dimensional influence mechanisms, they have not comprehensively explored the relationships between work–family conflict, work-to-family conflict, family-to-work conflict, and employee turnover intention, as a unified framework.

This study uses meta-analysis to determine the relationship between turnover intention and work–family conflict, as well as to understand its two conflict directions. These datasets examined are the body of existing empirical research. The research objective aims to answer two research questions:

(1) Do work–family conflict, work-to-family conflict, and family-to-work conflict significantly positively affect employees’ turnover intention?
(2) Do different gender, age, country, occupation type, and measurement methods significantly moderate the relationships between work–family conflict, work-to-family conflict, family-to-work conflict, and turnover intention?

## 2 Literature review and hypothesis development

### 2.1 Relationship between work–family conflict and turnover intention

Through extensive empirical research, scholars have found a significant positive correlation between work–family conflict and turnover intention (46, 47). Both work-to-family conflict and family-to-work conflict have significant predictive effects on turnover intention (48, 49). Sari et al. (15) pointed out that work–family conflict is significantly positively correlated with turnover intention. Kelloway et al. (50) found that both work-to-family conflict and family-to-work conflict have significant impacts on turnover intention. In the meta-analysis conducted by Mesmer-Magnus et al. (51), the concept of organizational withdrawal was introduced, and the research results were consistent with those of Kelloway. Aybas et al. (52) indicated that both work-to-family conflict and family-to-work conflict have significant positive effects on turnover intention.

Scholars have gradually delved into the study of work–family conflict and turnover intention in specific industries. Researchers conducted sampling surveys on ICT practitioners (53), hospital nurses (54), police officers (55), general corporate employees (56), female employees (57), and primary and secondary school teachers (58). They found that the more severe the work–family conflict, the stronger the employees’ turnover intention. Therefore, this study proposes the following hypotheses:

- Ha1: There is a significant positive correlation between work–family conflict and turnover intention.
- Ha2: There is a significant positive correlation between work-to-family conflict and turnover intention.
- Ha3: There is a significant positive correlation between family-to-work conflict and turnover intention.

### 2.2 Role of moderators

According to social role theory, factors such as physiological differences and socio-cultural influences, determine that men and women play different roles in organizational activities, thereby demonstrating distinct characteristics and cognitive abilities (59). During the work process, men tend to exhibit more proactive behaviors. For example, when there is a significant gap between current work and expectations or when better job opportunities arise, men’s intention to leave their jobs increases, and thus they are more likely to actually leave. In contrast, when job satisfaction is low, women tend to be more tolerant compared to men. After considering various factors, women generally do not propose leaving their jobs as readily as men (60). When exploring the impact of gender on the work-family relationship, social role theory was introduced, and empirical research confirmed that men have a higher work centrality and place more importance on their future career development as compared with women (61). Therefore, employees of different genders will have varying degrees of turnover intention when faced with work–family conflict.

Age, as a key demographic factor, significantly influences employees’ work attitudes and behavioral patterns (62). Relevant research indicates that age has a crucial impact on employees’ turnover intention (62, 63). According to embedded theory, individuals consider the loss of social status and material assets when leaving an organization or group (64). Therefore, older employees will consider the opportunity cost of leaving more carefully, thereby reducing their turnover intention (65). Younger employees are more willing to take risks and believe that new jobs will bring greater rewards (66). Moreover, compared to younger employees, older employees carry greater family responsibilities. In summary, employees of different age groups exhibit varying degrees of turnover intention when facing work–family conflict.

Through extensive empirical case studies, scholars have found differences based on cultural parameters. In the context of work–family conflict, job satisfaction has a greater impact on individualistic-dominated countries than on collectivistic-dominated countries. A comparison of American and Chinese families concluded that Chinese families consider family interests more important than personal interests, while American families regard family interests as equally important as personal interests (67).

Moreover, when analyzing the relationship between work–family conflict and turnover intention among subjects in different occupations, differences also appear. The relationship between work–family conflict and turnover intention among middle and senior managers in enterprises has been shown not to be significant (68). The correlation of research also varies among subjects in different occupations (69). Finally, the measurement tools used in different studies can also affect the research results. Therefore, this study proposes the following hypotheses:

- Hb1: Gender significantly moderates the relationship between work-to-family conflict, family-to-work conflict, and work–family conflict as three factors and turnover intention.
- Hb2: Age significantly moderates the relationship between work-to-family conflict, family-to-work conflict, and work–family conflict as three factors and turnover intention.
- Hb3: Country significantly moderates the relationship between work-to-family conflict, family-to-work conflict, and work–family conflict as three factors and turnover intention.
- Hb4: Occupational type significantly moderates the relationship between work-to-family conflict, family-to-work conflict, and work–family conflict as three factors and turnover intention.
- Hb5: Metrics Tool significantly moderates the relationship between work-to-family conflict, family-to-work conflict, and work–family conflict as three factors and turnover intention.

### 2.3 Theoretical framework

**Figure 1:**
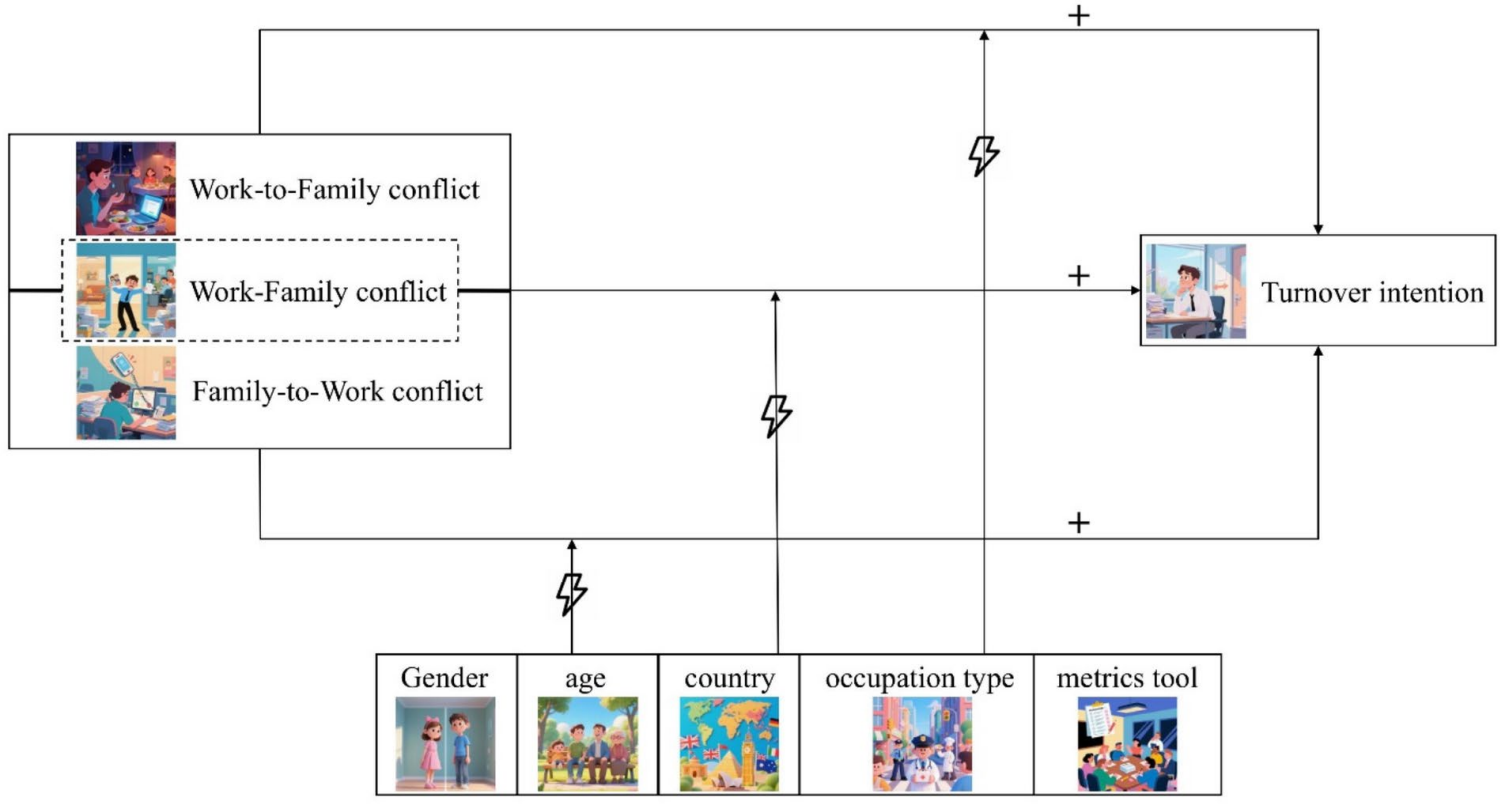
Theoretical model of meta-analysis.

Based on the above hypotheses, the following theoretical model diagram for meta-analysis is derived, as shown in Figure 1.

## 3 Meta-analytic research method

The meta-analysis is widely adopted to investigate employee turnover issues. Current meta-analyses mainly focus on factors affecting turnover intention, namely, leadership empowerment (70), psychological empowerment (71), career adaptability (72), worker characteristics and job attitudes (73), job satisfaction (74), organizational commitment (75), work environment indicators (76), and workplace incivility (77). The results determined by meta-analysis are validated more reliable and robust. Therefore, this study adopts the meta-analysis method to explore the relationship between work–family conflict and turnover intention.

Considering the increasing number of empirical studies on employee turnover intention, with a rising inconsistency in findings across studies, there is a need for a comprehensive quantitative analysis of research conclusions. Hedges and Olkin (78) pointed out that meta-analysis generates more stable overall estimates by combining effect sizes, such as correlation coefficient r, thereby avoiding ‘elective reporting bias.’ Through meta-analysis, dispersed studies can be quantitatively integrated, overcoming the contradictions in conclusions caused by insufficient sample sizes and design differences in single studies. This approach promises to resolve the issue of inconsistent findings.

**Figure 2:**
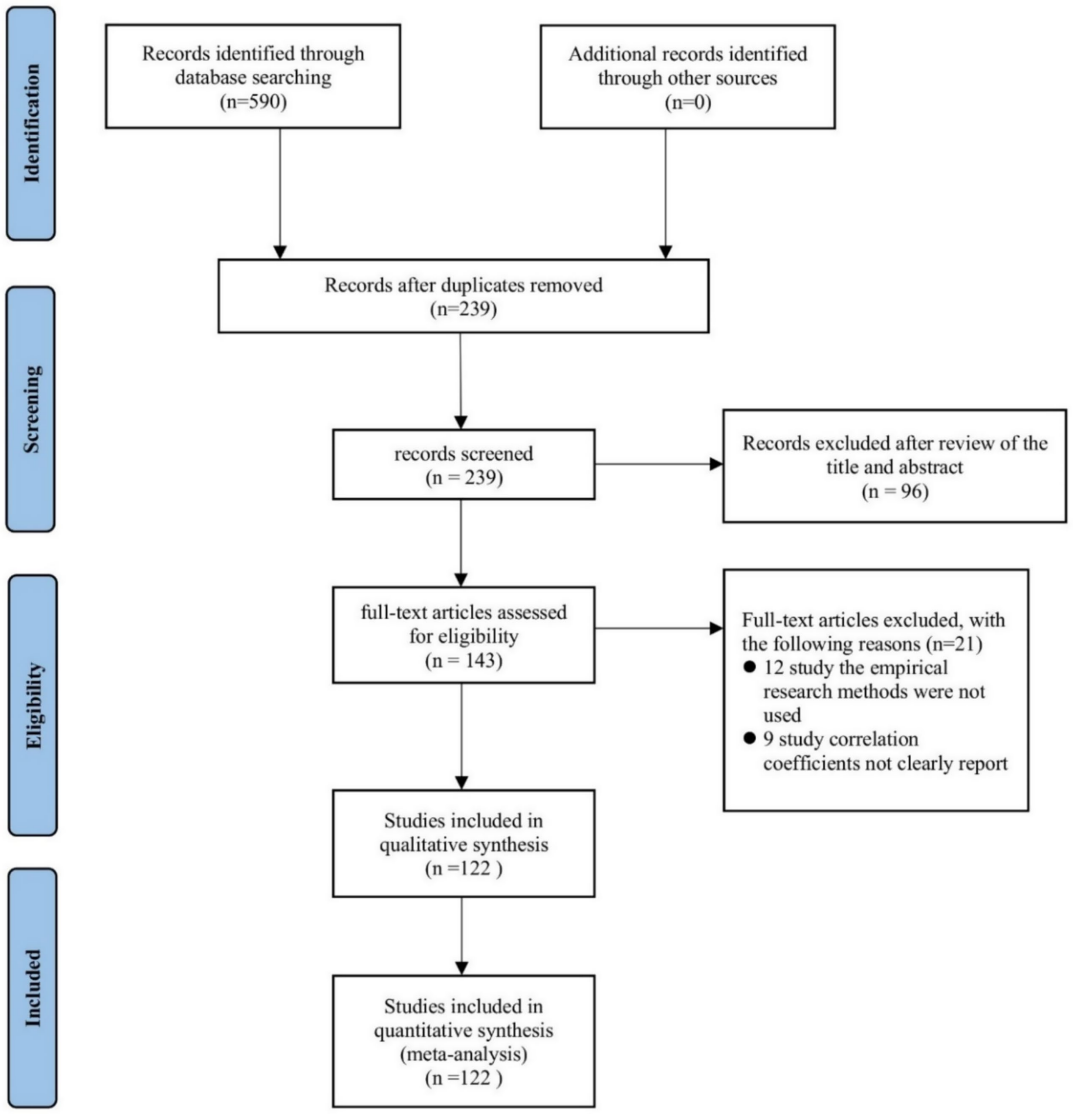
PRISMA flowchart for literature screening.

Therefore, this study adopts meta-analysis to conduct a scientific literature review of turnover intention research (77). Nevertheless, in order to minimize the outcome bias between work–family conflict and turnover intention, this study follows Stanley et al.’s meta-analysis procedures and statistical methods (79). The meta-analysis process includes literature search, screening, and coding, with specific steps, as shown in Figure 2.

### 3.1 Literature search and inclusion criteria

Firstly, in order to minimize publication bias, a comprehensive search was conducted across English literature databases, including Web of Science, Springer, Wiley, and Google Scholar, using the search terms “Turnover Intention,” “Intention to Quit,” “Work Family Conflict,” “Work–Family Conflict,” “Family–Work Conflict,” “Work Interference with Family,” and “Family Interference with Work.” The search was also conducted through Chinese literature databases, such as CNKI, VIP, and Wanfang Data, as Chinese scholars are highly active in this domain. Secondly, from 2010 to 2024, research on “work–family conflict” and “turnover intention” has been on the rise year by year. To ensure data reliability and continuity, we selected data spanning integer years, setting the literature search period from December 2010 to December 2024. Finally, to ensure rigor in the results, we applied the literature backtracking method, searching for unretrieved literature from the references of the already retrieved literature, in order to identify any additional significant literature.

Following a preliminary search, 416 English articles and 174 Chinese articles were retrieved, totaling 590 articles. To determine the final set of articles that need to be coded, the following criteria were developed for secondary screening.

(1) To avoid data duplication and redundancy, studies that were published at different stages, repeatedly published, or conducted using the same or overlapping samples were only included if they had larger sample sizes and greater detailed content, ensuring comprehensiveness and accuracy of the research (78). A total of 351 irrelevant articles were excluded.
(2) Only research literature containing dimensions related to turnover intention and work–family conflict was retained. This was to ensure the relevance of the research content. Consequently, a further 96 studies whose titles and abstracts do not meet the requirements were excluded.
(3) A further test was applied whereby only studies that invoked empirical research methods to verify the correlation between variables were retained. Specifically, literature reviews, case studies, and other theoretical research were removed, resulting in 12 further papers culled.
(4) This study utilizes the correlation coefficient between variables as the sole effect size, excluding studies that did not explicitly report the correlation coefficient between the independent variables and outcome variables involved. Thus, 9 more studies that did not provide effect sizes were rejected.

After a rigorous screening and elimination process, 122 independent empirical studies were finally selected, with an inclusion rate of 20.7%. Among these, 65 were in English and 57 were in Chinese. In terms of research content, 17 studies focused on work–family conflict, with 90 addressing work-to-family conflict, and 53 studies dealing with family-to-work conflict.

### 3.2 Document code

Using Excel 2021 software, systematic coding was conducted on the effective literature included in this meta-analysis. The coding process was undertaken in two steps. Firstly, descriptive items of the literature, covering key information such as study authors, publication date, and research subjects, were collected. Secondly, statistical values, including sample size, variable names involved in the study and their measurement dimensions, and correlation coefficients between different variables, were extracted.

Considering the complexity of the endeavor, this study divided the literature coding work into three stages. Firstly, coding content was clarified, which specifically covered author information, research objects, sample sizes, scale reliability, variable measurement methods and their specific dimensions, as well as correlation coefficients between variables and other key elements. Then, based on the previously determined coding framework, this study independently completed the coding work for valid literature and compiled preliminary coding summary tables accordingly, laying a data foundation for subsequent in-depth analysis. Finally, some literature from the preliminary coding table was randomly selected and carefully cross-checked with the original literature to ensure the accuracy and reliability of the coding. After further examination, a consensus was reached on all content of the coding, maintaining high consistency, and thus forming the final literature table. This process provided an accurate and reliable data platform necessary for the ensuing research analysis.

## 4 Meta-analysis results

Following the principles of meta-analysis proposed by Schmidt et al. (80), this study employs the data processing procedures involved in meta-analysis, including publication bias test, heterogeneity test, overall corrected weighted average effect size and associated statistical significance test. The corresponding data analysis was conducted using Comprehensive Meta Analysis software version 2 (CMA2.0).

### 4.1 Publication bias test

Publication bias test aims to evaluate the representativeness of selected literature, thereby avoiding result deviation caused by excessively high effect sizes.

**Figure 3:**
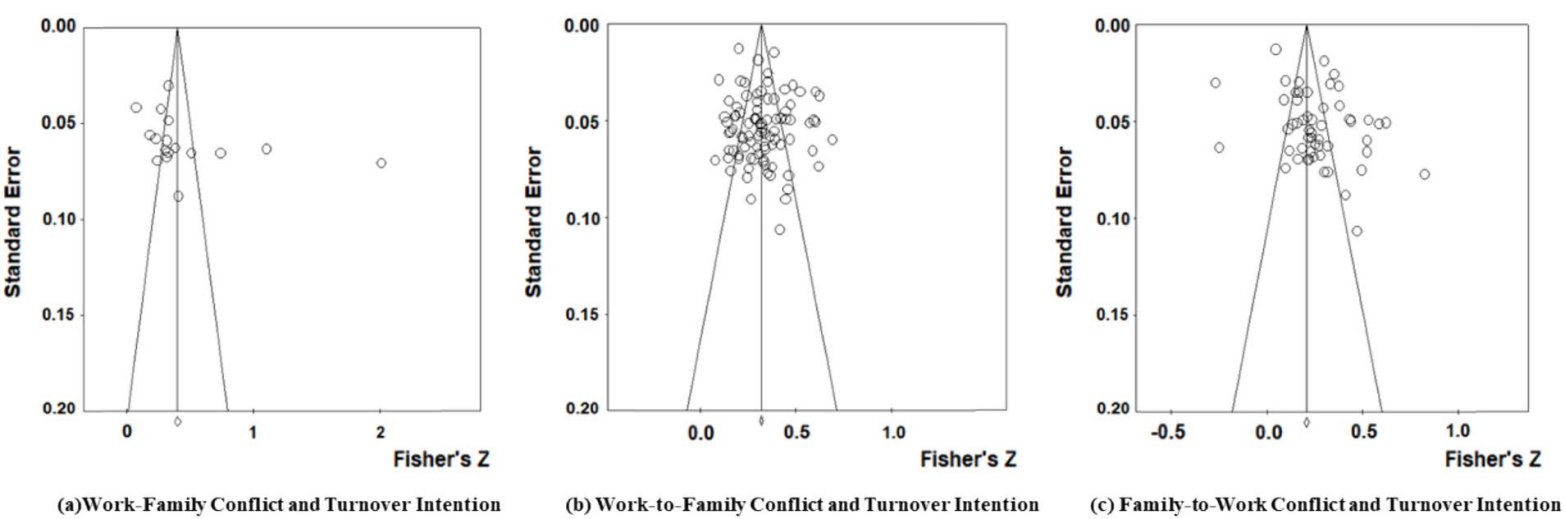
Funnel plot of three variables and turnover intention.

In general, funnel plots are used to preliminarily test whether there is publication bias in the coded data of effective literature (81). Based on the funnel plots shown in Figure 3, it is found that the effect sizes of the relationships between work–family conflict, work-to-family conflict, family-to-work conflict, and turnover intention are mainly concentrated at the top of the graph and show approximately central symmetric distribution patterns, indicating a low possibility of publication bias (82). According to Rosenthal’s Fail-safe N test criteria (83), the fail-safe coefficients (Nfs = 4,613, 19,438, 10,603) of hypotheses Ha1, Ha2, and Ha3 are significantly higher than the thresholds of 5 K + 10 (K represents the number of literature) (95, 460, 275), indicating a low probability of publication bias in this study. Based on both qualitative and quantitative analysis results, there is no publication bias in the literature coding data of this study, and the data have high reliability.

### 4.2 Heterogeneity test

Heterogeneity testing aims to determine the causes of differences between effect sizes and calculate the proportions of true error and random error. This study intends to conduct an in-depth analysis of multiple statistics of the effect size r between work–family conflict (one-dimensional), work-to-family conflict and family-to-work conflict (two-dimensional), and turnover intention, including Q statistic, I^2^ statistic, and Tau^2^ statistic.

Heterogeneity test results for the effect sizes of work–family conflict, work-to-family conflict, family-to-work conflict, and turnover intention are 780.844, 783.358, and 985.336, respectively, with *p* values all less than 0.001, indicating differences between independent samples, which may be caused by variations in literature sample sources, measurement methods, etc. The I^2^ values are 97.951, 88.639, and 94.723, respectively, indicating true differences of 97.951, 88.639, and 94.723%, respectively. The Tau^2^ values suggest that 14.6, 1.5, and 3.2% of the study result differences can be used for weight calculation, as shown in Table 1.

**Table 1:** Heterogeneity test results of effect sizes between antecedent variables and turnover intention.

Antecedent variables	K/piece	Heterogeneity				Tau-squared			
		Q	df	*p*	I^2^	Tau^2^	SE	Variance	Tau
Work-family	17	780.844	16	<0.001	97.951	0.146	0.061	0.004	0.383
Work-to-family	90	783.358	89	<0.001	88.639	0.015	0.004	0.000	0.121
Family-to-work	53	985.336	52	<0.001	94.723	0.032	0.011	0.000	0.178

Q, Degree of variation between different effect sizes; I^2^, Value is used to evaluate how much of the variation between effect sizes is caused by true differences; Tau^2^, Value quantifies the percentage of between-study differences in results weighted by study size.

It can be inferred that there is heterogeneity in the effect sizes between variables. According to Higgins et al.’s suggestion, the I^2^ values are divided into three levels: low (below 25%), medium (25–50%), and high (50–75%) to represent the degree of heterogeneity (84). If the heterogeneity is low, a fixed-effects model is more appropriate; if the heterogeneity is medium or high, a random-effects model is more appropriate (85). The results show that the I^2^ values are all higher than 75%, indicating high heterogeneity. Therefore, this study uses a random-effects model to analyze the impact of work–family conflict on turnover intention.

### 4.3 Main effect test

This study examines the K value, N value, r value, 95% confidence interval, Z value, and *p* value of the two-tailed test in the main effect test analysis, aiming to answer the research questions proposed by the three hypotheses. After screening, this study finally obtained 17, 90, and 53 empirical research articles on work–family conflict, work-to-family conflict, family-to-work conflict, and turnover intention, respectively. According to Cohen’s (86) classification criteria for effect size, small effect r = 0.1, medium effect r = 0.3, and large effect r = 0.5, as shown in Table 2. Among them, the effect size of work–family conflict and turnover intention (r = 0.446) is higher than the critical value of 0.3, belonging to a high degree, indicating a high correlation strength between the two, and the 95% confidence interval ([0.287, 0.581]) does not contain 0, which is statistically significant (*p* < 0.001). The effect size of work-to-family conflict and turnover intention (r = 0.318) is slightly higher than the critical value of 0.3, belonging to a medium effect, indicating that the correlation between the two has substantial significance, and the 95% confidence interval ([0.293, 0.343]) does not contain 0, which is statistically significant (*p* < 0.001). The effect size of family-to-work conflict and turnover intention (r = 0.261) is between 0.1 and 0.3, belonging to a small effect, reflecting the observability of this correlation in actual research, and the 95% confidence interval ([0.241, 0.307]) does not contain 0, which is statistically significant (*p* < 0.001). Therefore, it can be concluded that work–family conflict, work-to-family conflict, and family-to-work conflict have significant positive effects on turnover intention, and hypotheses Ha1, Ha2, and Ha3 are all validated.

**Table 2:** Total effect values between each antecedent variable and turnover intention.

Antecedent variables	Calculation model	K/piece	N	95%CI			Two-tailed test	
				r	Lower limits	Upper limits	Z	*p*
Work-family	Stochastic	17	5,752	0.446	0.287	0.581	5.101	<0.001
Work-to-family	Stochastic	90	51,110	0.318	0.293	0.343	23.498	<0.001
Family-to-work	Stochastic	53	33,779	0.261	0.214	0.307	10.434	<0.001

K, Number of independent samples in each study; N, Total sum of samples across all studies; r, True point estimation effect size between variables after correcting measurement errors.

### 4.4 Moderation effect test

In different studies, the correlation between work–family conflict, work-to-family conflict, family-to-work conflict, and turnover intention shows variations. This may be due to other potential variables playing a moderating role and influencing the relationship between variables. Therefore, based on the main effect analysis, this study examines the reasons for research finding differences across five aspects. Namely these are: gender, age, country, occupation type, and measurement tools.

For work–family conflict, four main results can be derived from the analysis, and details are as shown in Table 3. First, insufficient data on gender and age prevent analysis of the moderating effect of gender and age on the correlation between work–family conflict and turnover intention. Second, the moderating effect of different countries on the relationship between work–family conflict and turnover intention is significant, with India showing the highest correlation, followed by China, and then Pakistan. Third, the moderating effect of different occupation types on the relationship between work–family conflict and turnover intention is significant, with enterprise employees showing the highest correlation, and police showing the lowest. Fourth, different measurement tools have a significant moderating effect on the relationship be-tween work–family conflict and turnover intention, with Clark’s (102) scale showing the highest correlation, followed by Carlson et al. (23), and finally Netemeyer (87) indicating that different measurement tools and screening criteria can significantly moderate the correlation between variables.

**Table 3:** Moderating effect analysis of work–family conflict.

Moderator variable	Heterogeneity test			Class	K	Correlation	95%CI	
	QB	df	*p*				Lower limits	Upper limits
Country	387.32	2	<0.001	China	12	0.41	0.293	0.516
				India	1	0.965	0.954	0.973
				Pakistan	1	0.311	0.203	0.412
Occupation types	9.678	4	0.046	Enterprise staff	3	0.484	0.281	0.645
				Police	2	0.285	0.22	0.348
				Kindergarten teacher	2	0.323	0.277	0.367
Metrics tool	15.56	4	0.004	Carlson et al. (23)	9	0.419	0.251	0.562
				Clark (102)	1	0.474	0.368	0.568
				Netemeyer et al. (87)	1	0.323	0.268	0.376
				Wu Mingxia (103)	1	0.322	0.234	0.404

Regarding the work-to-family conflict, five important findings are revealed by the analysis, and details are as shown in Table 4. First, the moderating effect of gender was not significant (*p* > 0.05). Further analysis showed that the correlation coefficient between work-to-family conflict and turnover intention was 0.341 for males and 0.325 for females, with males slightly higher than females. Second, the moderating effect of age was not significant (*p* > 0.05). Further analysis found that the correlation coefficients between variables for ages 25–35, 35–45, and 45–55 were 0.320, 0.308, and 0.293, respectively. Third, the moderating effect of country was significant. Of these, China showed the highest correlation between work-to-family conflict and turnover intention, followed by the United States, and finally the Netherlands. This result indicates that the country of origin is an important factor, moderating the correlation between variables. Fourth, the moderating effect of occupation type was significant. Of these, preschool teachers showed the highest correlation between work-to-family conflict and turnover intention. The moderating effect of measurement tools was significant. Using the Netemeyer et al. (87) scale yielded the highest correlation between work-to-family conflict and turnover intention, followed by Carlson et al. (23), and finally Frone et al. (29). This indicates that different measurement tools and screening criteria are important factors affecting the correlation.

**Table 4:** Moderating effect analysis of work-to-family conflict.

Moderator variable	Heterogeneity test			Class	K	Correlation	95%CI	
	QB	df	p				Lower limits	Upper limits
Gender	0.049	1	>0.05	Male	7	0.341	0.21	0.461
				Female	7	0.325	0.257	0.39
Age	0.656	3	>0.05	25–35	9	0.32	0.22	0.414
				35–45	13	0.308	0.226	0.386
				45–55	6	0.293	0.16	0.416
Country	9.11	3	<0.001	China	47	0.308	0.277	0.338
				America	7	0.251	0.199	0.302
				Netherlands	3	0.19	0.072	0.302
Occupation types	43.963	14	<0.001	Nurse	14	0.34	0.242	0.431
				Enterprise staff	22	0.304	0.256	0.35
				Female staff	6	0.351	0.25	0.445
				Kindergarten teacher	3	0.377	0.305	0.444
				Hotel staff	3	0.344	0.224	0.454
				Construction staff	3	0.301	0.148	0.441
Metrics tool	8.027	3	0.045	Carlson et al. (23)	26	0.311	0.265	0.354
				Frone et al. (29)	3	0.194	0.106	0.279
				Netemeyer et al. (87)	33	0.33	0.281	0.377

For family-to-work conflict, five important findings are derived from the analysis, with the details shown in Table 5. First, the moderating effect of gender was not significant (*p* > 0.05). Further analysis revealed that the correlation coefficient between family-to-work conflict and turnover intention was 0.203 for males and 0.295 for females, indicating a slightly higher correlation for females compared to males. Second, the moderating effect of age was significant (*p* < 0.05). Further analysis showed that the correlation coefficients between family-to-work conflict and turnover intention for the age groups of 25–35, 35–45, and 45–55 were 0.350, 0.159, and 0.194, respectively. Among these, the highest correlation was found in the 25–35 age group, followed by the 45–55, and 35–45 age groups. Third, the moderating effect of nationality was significant, with Turkey showing the highest correlation between variables, followed by China and then the United States, indicating that nationality is an important factor affecting the correlation between variables. Fourth, the moderating effect of occupation type was significant, with construction workers showing the highest correlation between family-to-work conflict and turnover intention. Fifth, the moderating effect of measurement tools was significant, with the Netemeyer et al. (105, 106) scale showing the highest correlation between family-to-work conflict and turnover intention, followed by the Netemeyer (87) scale and Carlson (23) scale. This indicates that different measurement tools and screening criteria are important factors affecting the correlation between variables, as shown in Table 5. In summary, hypotheses Hb3, Hb4, and Hb5 were supported, while Hb1 was not supported, with hypothesis Hb2 gaining partial support.

**Table 5:** Moderating effect analysis of family-to-work conflict.

Moderator variable	Heterogeneity test			Class	K	Correlation	95%CI	
	QB	df	p				Lower limits	Upper limits
Gender	0.961	1	>0.05	Male	7	0.203	0.024	0.369
				Female	5	0.295	0.226	0.361
Age	8.885	2	0.012	25–35	6	0.35	0.252	0.44
				35–45	7	0.159	0.051	0.262
				45–55	2	0.194	0.139	0.248
Country	55.851	3	<0.001	America	5	0.174	0.132	0.216
				Turkey	2	0.523	0.447	0.591
				China	31	0.274	0.212	0.333
Occupation types	55.812	13	<0.001	Enterprise staff	15	0.245	0.171	0.316
				Nurse	8	0.244	0.152	0.331
				Construction staff	3	0.249	0.167	0.327
Metrics tool	47.787	6	<0.001	Carlson et al. (23)	15	0.28	0.193	0.362
				Netemeyer et al. (105, 106)	1	0.68	0.59	0.753
				Netemeyer et al. (87)	23	0.275	0.215	0.333

## 5 Discussion

Differences in sample characteristics and research methods across studies can lead to contradictory research findings. However, meta-analysis can help mitigate the impact of these differences (88). Therefore, meta-analytic results are considered more reliable and stable. The purpose of this paper is to conduct a meta-analysis on the relationship between work–family conflict and employees’ turnover intention. The analysis using random-effects meta-analysis procedures shows that work–family conflict, work-to-family conflict, and family-to-work conflict all have significant positive effects on turnover intention. What needs to be taken into consideration, however, is that different countries, occupation types, and measurement tools significantly moderate the relationships among these variables.

This study found a significant positive correlation between overall work–family conflict and turnover intention (r = 0.446), with both work-to-family conflict (r = 0.318) and family-to-work conflict (r = 0.261) showing statistically significant effects. This indicates that both work interference with family and family interference with work can increase employees’ turnover intention, while it is the former that has a stronger effect. From the perspective of Conservation of Resources Theory (89), when role obligations to work and family excessively deplete employees’ core resources, in time, energy, and emotion, employees tend to develop a turnover intention in the effort to alleviate conflict while conserving resources. For example, employees who cannot balance work and family due to high work pressure and frequent overtime, resulting in prolonged conflict, are more likely to develop turnover behavior. Longitudinal studies conducted by Smith et al. (104) confirmed the impact of work interfering with family, and its long-term driving effect on turnover intention. At the same time, the impact of family interfering with work is weaker due to situational fluctuations. According to research surveys (90), the growth rate and total working hours of employees significantly exceed family-related activity time, while working hours exhibit a trend of further encroaching on family life. Continuous work interference with family exacerbates psychological pressure through the “resource depletion” mechanism, ultimately leading to turnover intention under long-term accumulation effects. Therefore, work-to-family conflict has a greater impact on turnover intention as compared with family-to-work conflict.

The age factor only shows a moderating effect in the relationship between family-to-work conflict and turnover intention. Due to the lack of sufficient data, the moderating effect of gender in the relationship between work–family conflict and turnover intention cannot be analyzed. But Further analysis reveals that the correlation coefficient between family-to-work conflict and turnover intention was higher for women (r = 0.295) compared to men (r = 0.203), which again is consistent with social role theory. Traditional gender division of labor places greater family care responsibilities, such as childcare and housework, on women, making their psychological pressure more likely to translate into turnover intention when family demands interfere with work. Momin et al. (91) found that gender plays an important moderating role in the relationship between psychological capital and well-being, which provides that measuring variables such as psychological capital is beneficial to complement the research on the moderating effect of gender. Some studies have also pointed out that women have a stronger demand for flexible work arrangements, with more significant conflict mitigation effects. At the same time, men appear influenced by traditional ‘work-first’ role expectations that do not benefit significantly from flexible arrangements (92).

The moderating effect of age on family-to-work conflict was significant, with a higher correlation coefficient for employees aged 25–35 (r = 0.350) compared to those over 35. This indicates that work–family conflict is more likely to lead to turnover intention among younger employees. This can be attributed to differences in generational values. Growing up in a society where individualistic values are rising, younger employees emphasize self-realization and family life quality over traditional career loyalty (58). Research by Wan (93) and others has shown that younger employees value work-family balance more and consider family responsibilities as fundamental to life quality. When family responsibilities conflict with work, they are more inclined to maintain family harmony through turnover. Conversely, older employees, due to higher career embeddedness, such as seniority and networking, tend to endure or adjust role priorities (94).

In the study of five different moderating factors, the results showed that different countries, different occupational types, and different measurement tools all played a significant moderating role in the relationship between work–family conflict, work-to-family conflict, family-to-work conflict, and turnover intention.

The cultural differences between different countries significantly affected the correlation strength between the three types of conflicts and turnover intention. In countries dominated by collectivist cultures (such as China, r = 0.274), the correlation between family-to-work conflict and turnover intention was higher than in countries dominated by individualist cultures (such as the United States, r = 0.174). This difference can be explained by Hofstede’s individualism–collectivism dimension theory (95). In collectivist cultures, family interests take precedence over personal career development, and employees have a lower tolerance for family interference in work. By contrast, individualist cultures emphasize individual autonomy and career achievement, where employees are more inclined to cope with work conflicts by adjusting work strategies rather than by leaving their job (96).

Different occupational types also have a significant moderating effect on conflict effects. The correlation coefficient between work–family conflict and turnover intention among enterprise employees is the highest among different occupational types (r = 0.484). The reason for this lies in the fact that enterprise employees are required to devote as much time and energy as possible to work, making it difficult for them to meet family responsibilities. This can lead to increased conflict and thus increased turnover intention (97). Contrariwise, professional positions inculcated with a spirit of dedication, such as police officers, tend to have a deeper commitment to their chosen careers while holding their work to be personally meaningful (98). This sentiment makes them value their work, leaving them comfortable with investing time in their careers. They are thus less likely to have turnover intentions.

Similarly, the correlation coefficient between family-to-work conflict and turnover intention is the highest among construction industry employees (r = 0.249). The high mobility, irregular working hours, and lack of family-supportive supervisors in the construction industry make it particularly difficult for workers to balance work and family responsibilities (99). In the education sector, preschool teachers have a prominent work-to-family conflict effect (r = 0.377). Preschool teachers need to spend more time and energy on teaching and caring for young children, which may lead them to neglect their own families’ needs for time and energy. Work overload causes pre-school teachers to still need to handle lesson preparation, parent communication, and other tasks after work, squeezing family time and leading to increased work-to-family conflict, resulting in turnover intention (100).

Of the different measurement tools, the scales developed by Carlson et al. (23) and Netemeyer et al. (87) are most commonly used. Both revealed the most significant moderating effect to be between family-to-work conflict and turnover intention. However, the Carlson scale had a greater impact on the moderating effect between work-to-family conflict, family-to-work conflict, and turnover intention. According to the findings of Carmines and Zeller (101), measurement tools shape empirical results through construct coverage, item quality, and cultural adaptability, indicating that differences in scale structure design, reliability, and cultural adaptability can lead to differences in correlation coefficients. The six-dimensional subdivision screening criteria and form decomposition of the Carlson et al. (23) scale enhanced the sensitivity of specific correlations, explaining its higher correlation. The bidirectional structure of the Netemeyer et al. (87) scale prioritizes comprehensive screening criteria and simplicity, leading to the ‘aggregation and dilution’ of correlation.

## 6 Conclusion

This study reviews and analyzes the current research status of work–family conflict and turnover intention both domestically and internationally. In so doing, it points out the limitations of existing research on work–family conflict and turnover intention. To address the shortcomings in current turnover intention research, this study employs a meta-analysis method to quantitatively analyze the correlation between work–family conflict (r = 0.446), work-to-family conflict (r = 0.318), family-to-work conflict (r = 0.261), and turnover intention. The results showed that all these conflicts have a significant positive impact on turnover intention.

However, different countries, occupation types, and measurement tools significantly moderate the correlation between work–family conflict, work-to-family conflict, family-to-work conflict, and turnover intention. In addition, age was also confirmed to significantly moderate the correlation between family-to-work conflict and turnover intention. The findings of this research provide theoretical support for organizations aiming to develop and improve work-family policies in order to retain valued employees. There are two major implications. First, the implementation of differentiated career management should be considered. For high-conflict occupations, such as nursing and construction, it is necessary to optimize shift systems and provide psychological counseling services. In the education sector, in order to reduce conflict perception, there ought to be a reduction in the administrative burden combined with teacher autonomy. Second, work-family balance policies must be developed in ways that fit local cultural characteristics. Based on cultural dimensions, such as individualism/collectivism, enterprises should provide employees with organizational support measures that align with local culture. In collectivist culture-dominated countries, such as China, family support policies, such as flexible working hours and childcare support, can be strengthened to reduce the interference of family responsibilities on work. In individualistic culture-dominated countries, such as the United States, more emphasis should be placed on career development and work autonomy support, such as flexible remote work and personalized career planning. This would help employees regulate psychological pressure through cultural adaptation, thus reducing turnover intention caused by work–family conflict.

However, this study still has limitations. First, the gender moderating effect in the relationship between work–family conflict and turnover intention was not sufficiently supported by data. Possibly this is limited by the ambiguity of gender classifications in the literature, where some studies did not clearly report gender distribution. Second, the age moderating effect was only reflected in the family-to-work conflict dimension, thus lacking general significance. Third, the comparison samples of different countries’ moderating effects were concentrated in only a few countries (China, n = 59), with a lack of studies from a diverse range of national backgrounds. This will affect the generalizability of conclusions.

Future research can add sensitivity analysis of moderating factors. By evaluating the sensitivity of gender factors to variables, the actual impact of gender as a moderating factor can be reconsidered to make up for the lack of gender factors in data. Despite scientific meta-analytic screening, the existing databases still exhibit a structural imbalance: developing countries, China in particular, have significantly more research outputs in the field of work–family conflict compared to other nations. This phenomenon likely reflects that the practical urgency of this issue in developing countries has driven academic attention. For instance, China’s needs for industrial transformation and rapid economic development have stimulated the growth of research in the realm of work–family conflict. Future research can combine cross-cultural comparisons and dynamic tracking design, expand the language range of data collection to incorporate more national background studies, which is conducive to more prominently addressing geographical biases, deeply exploring the cultural adaptation mechanism of work–family conflict, and exploring potential mediating variables such as perceived organizational support and psychological capital to improve the theoretical framework. In addition, given the high-conflict characteristics of the construction industry, it is recommended to combine the guidance of psychological capital and organizational support systems to provide a way to reduce the intention to leave in highly conflicted environments.

## Data Availability

The original contributions presented in the study are included in the article, further inquiries can be directed to the corresponding author/s.

## References

[1] Dogru T McGinley S Sharma A Isık C Hanks L. Employee turnover dynamics in the hospitality industry vs. the overall economy. Tour Manag. (2023) 99:104783. doi: 10.1016/j.tourman.2023.104783

[2] Loh MY Dollard MF Friebel A. Economic costs of poor PSC manifest in sickness absence and voluntary turnover. Econ Labour Relat Rev. (2024) 35:635–48. doi: 10.1017/elr.2024.42

[3] Akhtar MS Salleh LM Ghafar NH Khurro MA Mehmood SA. Conceptualizing the impact of perceived organizational support and psychological contract fulfillment on employees’ paradoxical intentions of stay and leave. Int J Eng Technol UAE. (2018) 7:9–14. doi: 10.14419/ijet.v7i2.5.10045

[4] Eckardt R Skaggs BC Youndt M. Turnover and knowledge loss: an examination of the differential impact of production manager and worker turnover in service and manufacturing firms. J Manage Stud. (2014) 51:1025–57. doi: 10.1111/joms.12096

[5] Griffeth RW Hom PW Gaertner S. A Meta-analysis of antecedents and correlates of employee turnover: update, moderator tests, and research implications for the next millennium. J Manag. (2000) 26:463–88. doi: 10.1177/014920630002600305, PMID: 40778240

[6] Moen P Kelly EL Lee S-R Oakes JM Fan W Bray J . Can a flexibility/support initiative re-duce turnover intentions and exits? Results from the work, family, and health network. Soc Probl. (2017) 64:53–85. doi: 10.1093/socpro/spw033

[7] Halawi AH. Stimuli and effect of the intention to leave the organization. Eur Sci J. (2014) 10:123–35.

[8] Zhang Y. A review of employee turnover influence factor and countermeasure. J Hum Resour Sustain Stud. (2016) 4:85–91. doi: 10.4236/jhrss.2016.42010

[9] Zhang X Chen X Dai L Long Y Wang Z Shindo K. The effect of work stress on turnover intention amongst family doctors: a conditional process analysis. Int J Health Plann Manag. (2023) 38:1300–13. doi: 10.1002/hpm.3652, PMID: 37164642

[10] Lu Y Hu X-M Huang X-L Zhuang X-D Guo P Feng L-F . The relationship between job satisfaction, work stress, work–family conflict, and turnover intention among physicians in Guangdong, China: a cross-sectional study. BMJ Open. (2017) 7:e014894. doi: 10.1136/bmjopen-2016-014894, PMID: 28501813 PMC5566636

[11] Pennbrant S Dåderman A. Job demands, work engagement and job turnover intentions among registered nurses: explained by work-family private life inference. Work. (2021) 68:1157–69. doi: 10.3233/WOR-213445, PMID: 33867375

[12] Rhee M Park SK Lee C. Pathways from workplace flexibility to turnover intention: role of work–family conflict, family–work conflict, and job satisfaction. Int J Soc Welfare. (2020) 29:51–61. doi: 10.1111/ijsw.12382

[13] Le H Lee J Nielsen I Nguyen TLA. Turnover intentions: the roles of job satisfaction and family support. Pers Rev. (2023) 52:2209–28. doi: 10.1108/PR-08-2021-0582

[14] Senapaty S Venugopal P. When do personal factors make autonomy motivational orientation worthwhile? A case of turnover intentions. J Hum Values. (2023) 29:296–304. doi: 10.1177/09716858231172590

[15] Sari SY Yenni Z Aima MH. Determinants of turnover intention: job satisfaction, employee retention, work-family conflict and organisational commitment. Int Rev Manag Mark. (2024) 14:26–36. doi: 10.32479/irmm.16979

[16] Poulose J Sharma V. Exploring the mediating role of job and life satisfaction between work–family conflict, family–work conflict and turnover intention. Evid Based HRM. (2024) 13:466–83. doi: 10.1108/EBHRM-04-2023-0091

[17] Reimann M Schulz F Marx CK Lükemann L. The family side of work-family conflict: a literature review of antecedents and consequences. J Fam Res. (2022) 34:1010–32. doi: 10.20377/jfr-859

[18] Greenhaus JH Beutell NJ. Sources of conflict between work and family roles. Acad Manag Rev. (1985) 10:76. doi: 10.2307/258214

[19] Mukanzi CM Senaji TA. Work–family conflict and employee commitment: the moderating effect of perceived managerial support. SAGE Open. (2017) 7:2158244017725794. doi: 10.1177/2158244017725794

[20] Rotondo DM Carlson DS Kincaid JF. Coping with multiple dimensions of work-family conflict. Pers Rev. (2003) 32:275–96. doi: 10.1108/00483480310467606

[21] Byron K. A meta-analytic review of work–family conflict and its antecedents. J Vocat Behav. (2005) 67:169–98. doi: 10.1016/j.jvb.2004.08.009

[22] Frone MR Yardley JK Markel KS. Developing and testing an integrative model of the work–family interface. J Vocat Behav. (1997) 50:145–67. doi: 10.1006/jvbe.1996.1577

[23] Carlson DS Kacmar KM Williams LJ. Construction and initial validation of a multidimensional measure of work–family conflict. J Vocat Behav. (2000) 56:249–76. doi: 10.1006/jvbe.1999.1713

[24] Wang C Chang X Zhou Y Zhu H. How do work-family practices influence employee work-family conflict? Moderations of commitment-based HRM and human capital. Pers Rev. (2024) 53:2209–32. doi: 10.1108/PR-08-2021-0554

[25] Greenhaus JH Parasuraman S Collins KM. Career involvement and family involvement as moderators of relationships between work–family conflict and withdrawal from a profession. J Occup Health Psychol. (2001) 6:91–100. doi: 10.1037/1076-8998.6.2.91, PMID: 11326728

[26] Peeters MCW Montgomery AJ Bakker AB Schaufeli WB. Balancing work and home: how job and home demands are related to burnout. Int J Stress Manag. (2005) 12:43–61. doi: 10.1037/1072-5245.12.1.43

[27] Kirchmeyer C Cohen A. Different strategies for managing the work non-work interface: a test for unique pathways to work outcomes. Work Stress. (1999) 13:59–73. doi: 10.1080/026783799296192

[28] Shaffer MA Harrison DA Gilley KM Luk DM. Struggling for balance amid turbulence on international assignments: work–family conflict, support and commitment. J Manag. (2001) 27:99–121. doi: 10.1177/014920630102700106, PMID: 40778240

[29] Frone MR Russell M Cooper ML. Antecedents and outcomes of work-family conflict: testing a model of the work-family interface. J Appl Psychol. (1992) 77:65–78. doi: 10.1037/0021-9010.77.1.65, PMID: 1556042

[30] Eby LT Casper WJ Lockwood A Bordeaux C Brinley A. Work and family research in IO/OB: content analysis and review of the literature (1980–2002). J Vocat Behav. (2005) 66:124–97. doi: 10.1016/j.jvb.2003.11.003

[31] Xie Q Xie N Yang G. Do family affairs matter? Work–family conflict and safety behavior of construction workers. J Manag Eng. (2022) 38:04021074. doi: 10.1061/(ASCE)ME.1943-5479.0000977

[32] Cardenas RA Major DA Bernas KH. Exploring work and family distractions: antecedents and outcomes. Int J Stress Manag. (2004) 11:346–65. doi: 10.1037/1072-5245.11.4.346

[33] Cerrato J Cifre E. Gender inequality in household chores and work-family conflict. Front Psychol. (2018) 9:1330. doi: 10.3389/fpsyg.2018.01330, PMID: 30123153 PMC6086200

[34] Yoo J. Gender role ideology, work–family conflict, family–work conflict, and marital satisfaction among Korean dual-earner couples. J Fam Issues. (2022) 43:1520–35. doi: 10.1177/0192513X211026966

[35] Rubio C Osca A Recio P Urien B Peiró JM. Work-family conflict, self-efficacy, and emotional exhaustion: a test of longitudinal effects. Rev Psicol Trab Organ. (2015) 31:147–54. doi: 10.1016/j.rpto.2015.06.004

[36] Rani Thanacoody P Bartram T Casimir G. The effects of burnout and supervisory social support on the relationship between work-family conflict and intention to leave: a study of Australian cancer workers. J Health Organ Manag. (2009) 23:53–69. doi: 10.1108/14777260910942551, PMID: 19455878

[37] Huang M-H Cheng Z-H. The effects of inter-role conflicts on turnover intention among frontline service providers: does gender matter? Serv Ind J. (2012) 32:367–81. doi: 10.1080/02642069.2010.545391

[38] Purwayoga PVS Dharmanegara IBA Yasa PNS. Mediating role of work engagement and emotional exhaustion in the effect of work-family conflict on female workers’ turnover intention. Int J Acad Res Bus Soc Sci. (2019) 9:176–90. doi: 10.6007/IJARBSS/v9-i7/6101

[39] Li X Guo Y Zhou S. Chinese preschool teachers’ income, work-family conflict, organizational commitment, and turnover intention: a serial mediation model. Child Youth Serv Rev. (2021) 128:106005. doi: 10.1016/j.childyouth.2021.106005

[40] Jia CX Li JC. Work-family conflict, burnout, and turnover intention among Chinese social workers: the moderating role of work support. J Soc Serv Res. (2022) 48:12–27. doi: 10.1080/01488376.2021.1942393

[41] Yildiz B Yildiz H Ayaz Arda O. Relationship between work–family conflict and turnover intention in nurses: a meta-analytic review. J Adv Nurs. (2021) 77:3317–30. doi: 10.1111/jan.14846, PMID: 33855744

[42] Blomme RJ Van Rheede A Tromp DM. Work-family conflict as a cause for turnover intentions in the hospitality industry. Tour Hosp Res. (2010) 10:269–85. doi: 10.1057/thr.2010.15

[43] Wang F Wang Z. A mediation moderation model between work–family conflict and turnover intention among public and private kindergarten school teachers in China. J Organ Chang Manage. (2024) 37:116–32. doi: 10.1108/JOCM-04-2023-0137

[44] Wilkinson S Haar J. Smartdevice use in a COVID-19 world: exploring work–family conflict and turnover intentions. Asia Pac J Hum Resour. (2023) 61:981–1007. doi: 10.1111/1744-7941.12370

[45] Li D Li X Wang L Wang G Newton C. Work–family conflict in-fluences the relationship between family embeddedness and turnover intention. Soc Behav Pers. (2019) 47:1–13. doi: 10.2224/sbp.7640

[46] Chen I Brown R Bowers BJ Chang W. Work-to-family conflict as a mediator of the relationship between job satisfaction and turnover intention. J Adv Nurs. (2015) 71:2350–63. doi: 10.1111/jan.12706, PMID: 26043649

[47] Huang HI. Understanding culinary arts workers: locus of control, job satisfaction, work stress and turnover intention. J Foodserv Bus Res. (2006) 9:151–68. doi: 10.1300/J369v09n02_09

[48] Farhadi P Sharifian R Feili A Shokrpour N. The effects of supervisors’ supportive role, job stress, and work-family conflicts on the nurses’ attitudes. Health Care Manag. (2013) 32:107–22. doi: 10.1097/HCM.0b013e31828ef5e7, PMID: 23629033

[49] Wen Z Xu J Yu J Huang X Ni Y. Effects of work-family conflict on turnover intention among primary medical staff in Huaihai economic zone: a mediation model through burnout. Front Psych. (2023) 14:1238315. doi: 10.3389/fpsyt.2023.1238315, PMID: 37817834 PMC10561281

[50] Kelloway EK Gottlieb BH Barham L. The source, nature, and direction of work and family conflict: a longitudinal investigation. J Occup Health Psychol. (1999) 4:337–46. doi: 10.1037/1076-8998.4.4.337, PMID: 10526838

[51] Mesmer-Magnus JR Viswesvaran C. Convergence between measures of work-to-family and family-to-work conflict: a meta-analytic examination. J Vocat Behav. (2005) 67:215–32. doi: 10.1016/j.jvb.2004.05.004

[52] Aybas M Özçelik G Uyargil C. Can decent work explain employee-level outcomes? The roles of work–family and family–work conflict. Sustainability. (2022) 14:11488. doi: 10.3390/su141811488

[53] Lee LM Gan SW Chia YS. Investigating the effects of work-family conflict on turnover intention of ICT employees in Malaysia. Makara Hum Behav Stud Asia. (2023) 27:1–10. doi: 10.7454/hubs.asia.1131022

[54] Mafula D Arifin H Chen R Sung C-M Lee C-K Chiang K-J . Prevalence and moderating factors of turnover rate and turnover intention among nurses worldwide: a meta-analysis. J Nurs Regul. (2025) 15:20–36. doi: 10.1016/S2155-8256(25)00031-6

[55] Li JCM Cheung C Sun IY Cheung Y Zhu S. Work–family conflicts, stress, and turnover intention among Hong Kong police officers amid the COVID-19 pandemic. Police Q. (2022) 25:281–309. doi: 10.1177/10986111211034777, PMID: 36065392 PMC9361033

[56] Nohe C Sonntag K. Work–family conflict, social support, and turnover intentions: a longitudinal study. J Vocat Behav. (2014) 85:1–12. doi: 10.1016/j.jvb.2014.03.007

[57] Rasheed M Iqbal S Mustafa F. Work-family conflict and female employees’ turnover intentions. Gend Manag. (2018) 33:636–53. doi: 10.1108/GM-09-2017-0112

[58] Li X Chen X Gao D. Influence of work-family conflict on turnover intention of primary and secondary school teachers: serial mediating role of psychological contract and job satisfaction. Front Psych. (2022) 13:869344. doi: 10.3389/fpsyt.2022.869344, PMID: 35558430 PMC9086593

[59] Williams JE Best DL. Measuring sex stereotypes: A multination study. Newbury Park, California, USA: Sage Publications, Inc (1990).

[60] Stewart SM Bing MN Gruys ML Helford MC. Men, women, and perceptions of work environments, organizational commitment, and turnover intentions. J Bus Public Aff. (2007) 1:1–21.

[61] Sweet S Sarkisian N Matz-Costa C Pitt-Catsouphes M. Are women less career centric than men? Structure, culture, and identity investments. Community Work Family. (2016) 19:481–500. doi: 10.1080/13668803.2015.1078287

[62] Ng TWH Feldman DC. The relationships of age with job attitudes: a meta-analysis. Pers Psychol. (2010) 63:677–718. doi: 10.1111/j.1744-6570.2010.01184.x

[63] Christensen JO Knardahl S. “I’m too old for this!”: a prospective, multilevel study of job characteristics, age, and turnover intention. Front Psychol. (2022) 13:1015313. doi: 10.3389/fpsyg.2022.1015313, PMID: 36507023 PMC9730520

[64] Rubenstein AL Kammeyer-Mueller JD Wang M Thundiyil TG. “Embedded” at hire? Predicting the voluntary and involuntary turnover of new employees. J Organ Behav. (2019) 40:342–59. doi: 10.1002/job.2335

[65] Peltokorpi V Allen DG Froese F. Organizational embeddedness, turnover intentions, and voluntary turnover: the moderating effects of employee demographic characteristics and value orientations. J Organ Behav. (2015) 36:292–312. doi: 10.1002/job.1981

[66] Huynh LN. What factors do workers consider when changing jobs? Mon Labor Rev. (2024) 147:1–14.

[67] Wang YL He SZ. A review of work-family conflict from the cross-cultural perspective. Manag Rev. (2008) 20:21–7.

[68] Zhang M Griffeth RW Fried DD. Work-family conflict and individual consequences. J Managerial Psychol. (2012) 27:696–713. doi: 10.1108/02683941211259520

[69] Zwan RVD Yerkes MA Besamusca JW Kruyen PM Remery CL. What role do occupational differences play in subjective working conditions throughout the COVID-19 pandemic? Sociol Inq. (2024) 94:673–89. doi: 10.1111/soin.12574

[70] Zhang J Lin S Liu S Zhang Y Li H. Empowering leadership and leadership effectiveness: a meta-analytic examination. Adv Psychol Sci. (2021) 29:1576–98. doi: 10.3724/SP.J.1042.2021.01576

[71] Llorente-Alonso M García-Ael C Topa G. A meta-analysis of psychological empowerment: antecedents, organizational outcomes, and moderating variables. Curr Psychol. (2024) 43:1759–84. doi: 10.1007/s12144-023-04369-8

[72] Rudolph CW Lavigne KN Zacher H. Career adaptability: a me-ta-analysis of relationships with measures of adaptivity, adapting responses, and adaptation results. J Vocat Behav. (2017) 98:17–34. doi: 10.1016/j.jvb.2016.09.002

[73] Morrison DL Savery LK. The role of worker and job characteristics on turnover intentions. Int J Hum Factors Manuf. (1996) 6:263–79. doi: 10.1002/(SICI)1522-7111(199622)6:3<>3.3.CO;2-D

[74] Özkan AH. A meta-analysis of the variables related to turnover intention among IT personnel. Kybernetes. (2022) 51:1584–600. doi: 10.1108/K-02-2021-0098

[75] Ozkan AH Elci M Karabay ME Kitapci H Garip C. Antecedents of turnover intention: a meta-analysis study in the United States. E+M Ekonomie a Management. (2020) 23:93–110. doi: 10.15240/TUL/001/2020-1-007

[76] Kim H Kao D. A meta-analysis of turnover intention predictors among U.S. child welfare workers. Child Youth Serv Rev. (2014) 47:214–23. doi: 10.1016/j.childyouth.2014.09.015

[77] Namin BH Øgaard T Røislien J. Workplace incivility and turn-over intention in organizations: a meta-analytic review. Int J Environ Res Public Health. (2021) 19:25. doi: 10.3390/ijerph19010025, PMID: 35010292 PMC8751201

[78] Hedges LV Olkin I. Statistical methods for meta-analysis. 1st ed. Orlando, Florida, USA: Academic Press (1985).

[79] Havránek T Stanley TD Doucouliagos H Bom P Geyer-Klingeberg J Iwasaki I . Reporting guide-lines for meta-analysis in economics. J Econ Surv. (2020) 34:469–75. doi: 10.1111/joes.12363

[80] Hunter JE Schmidt FL. Methods of meta-analysis: Correcting error and bias in research findings. Thousand Oaks, California, USA: Sage Publications (2004).

[81] Mavridis D Salanti G. How to assess publication bias: funnel plot, trim-and-fill method and selection models. Evid Based Ment Health. (2014) 17:30–48. doi: 10.1136/eb-2013-101699, PMID: 24477535 PMC13404685

[82] Xu Y Li CP. The relationship between leadership styles and engagement: a meta-analysis. Acta Psychol Sin. (2019) 51:693–706. doi: 10.3724/SP.J.1042.2019.01363

[83] Rosenthal R. The file drawer problem and tolerance for null results. Psychol Bull. (1979) 86:638–41. doi: 10.1037/0033-2909.86.3.638

[84] Higgins JPT. Measuring inconsistency in meta-analyses. Br Med J. (2003) 327:557–60. doi: 10.1136/bmj.327.7414.557, PMID: 12958120 PMC192859

[85] Borenstein M Hedges LV Higgins JP Rothstein HR. Intro-duction to meta-analysis. Chichester, UK: John Wiley & Sons (2021).

[86] Cohen J. Statistical power analysis for the behavioral sciences. London: Routledge (2013).

[87] Netemeyer RG Boles JS McMurrian R. Development and validation of work-family conflict and family-work conflict scales. J Appl Psychol. (1996) 81:400–10. doi: 10.1037/0021-9010.81.4.400

[88] Lilienthal J Sturtz S Schürmann C Maiworm M Röver C Friede T . Bayesian random-effects meta-analysis with empirical heterogeneity priors for application in health technology assessment with very few studies. Res Synth Methods. (2024) 15:275–87. doi: 10.1002/jrsm.1685, PMID: 38152969

[89] Hobfoll SE. Conservation of resources: a new attempt at conceptualizing stress. Am Psychol. (1989) 44:513–24. doi: 10.1037/0003-066X.44.3.513, PMID: 2648906

[90] OECD. OECD Employment Outlook 2021: Navigating the COVID-19 Crisis and Recovery. Paris, France: OECD (2021).

[91] Momin MM Rolla KP. Exploring the multi-faceted nature of wellbeing across genders: evaluating the antecedence of psychological capital and life satisfaction. Gend Issues. (2024) 41:11. doi: 10.1007/s12147-024-09328-6

[92] Gunaprasida N Wibowo A. The effect of work-family conflict and flexible work arrangement on turnover intention: do female and male employees differ? J Siasat Bisnis. (2019) 23:27–36. doi: 10.20885/jsb.vol23.iss1.art3

[93] Wan M(M) Shaffer MA Singh R Zhang Y. Spoiling for a fight: a relational model of daily work-family balance satisfaction. J Occup Organ Psychol. (2022) 95:60–89. doi: 10.1111/joop.12368

[94] Mitchell TR Holtom BC Lee TW Sablynski CJ Erez M. Why people stay: using job embeddedness to predict voluntary turnover. Acad Manag J. (2001) 44:1102–21. doi: 10.5465/3069391

[95] Hofstede G. Culture’s consequences: Comparing values, behaviors, institutions and organizations across nations. Thousand Oaks, California, USA: Sage Publications (2001).

[96] Spector PE Allen TD Poelmans SAY Lapierre LM Cooper CL Michael O . Cross-national differences in relationships of work demands, job satisfaction, and turnover intentions with work–family conflict. Pers Psychol. (2007) 60:805–35. doi: 10.1111/j.1744-6570.2007.00092.x

[97] Shang S Chan XW Liu X. Work–life conflict in China: a Confucian cultural perspective In: Adisa TA Gbadamosi G, editors. Work-life Interface. Cham: Springer International Publishing (2021). 249–84.

[98] Lo V. Police stress in Hong Kong: a qualitative study. Int J Police Sci Manag. (2012) 14:113–23.

[99] Liu B Wang Q Wu G Zheng J Li L. How family-supportive supervisor affect Chinese construction workers’ work-family conflict and turnover intention: investigating the moderating role of work and family identity salience. Constr Manag Econ. (2020) 38:807–23. doi: 10.1080/01446193.2020.1748892

[100] Zhou S Li X Gao B. Family/friends support, work-family conflict, organizational commitment, and turnover intention in young preschool teachers in China: a serial mediation model. Child Youth Serv Rev. (2020) 113:104997. doi: 10.1016/j.childyouth.2020.104997

[101] Carmines EG Zeller RA. Reliability and validity assessment Thousand Oaks, California, USA: Sage Publications (1979).

[102] Clark SC. Work Cultures and Work/Family Balance. J Vocat Behav (2001) 58:348–365. doi: 10.1006/jvbe.2000.1759

[103] Ming-xia W Da-jun Z Xu C Lin Y Cheng G. The Measure of the Work-family Conflict of Chinese Elementary and Secondary School Teachers[J]. Psychological Development and Education. (2009) 25:120–127.

[104] Smith CE Wayne JH Matthews RA Lance CE Griggs TL Pattie MW. Stability and change in levels of work–family conflict: A multi‐study, longitudinal investigation. J Occup Organ Psychol (2022) 95:1–35. doi: 10.1111/joop.12372

[105] Netemeyer RG Brashear-Alejandro T Boles JS. A Cross-National Model of Job-Related Outcomes of Work Role and Family Role Variables: A Retail Sales Context. J Acad Mark Sci (2004) 32:49–60. doi: 10.1177/0092070303259128

[106] Netemeyer RG Maxham JG Pullig C. Conflicts in the Work–Family Interface: Links to Job Stress, Customer Service Employee Performance, and Customer Purchase Intent. J Mark (2005) 69:130–143. doi: 10.1509/jmkg.69.2.130.60758

